# Religion and Spirituality Measures in Dementia: An Integrative Review of the Literature

**DOI:** 10.1093/geroni/igab046.2759

**Published:** 2021-12-17

**Authors:** Katherine Britt, Gayle Acton, Jung Kwak

**Affiliations:** 1 University of Texas at Austin, Austin, Texas, United States; 2 University of Texas at Austin, The, Austin, Texas, United States; 3 The University of Texas at Austin, Austin, Texas, United States

## Abstract

Literature documents positive effects of religion and spirituality on health including improved cognitive function, quality of life, and well-being. Extant research suggests that persons living with dementia (PWD) are more likely to have spiritual needs and rely on others to support their spiritual well-being than those without dementia. However, spiritual care is absent or minimally present in dementia care. To effectively address spiritual needs of PWDs, accurate screening and assessment is critical. We conducted an integrative review of the current literature on measures of religion and spiritualty for PWDs by searching five databases (ATLA Religion, CINAHL, PsychInfo, PubMed, and SocIndex) and identified 14 studies that were peer-reviewed original research articles focusing on assessment of religion/spirituality among PWDs and published between 2000-2020 in English. Most were conducted in Europe (n=7), included PWD in mild stage (n=68) from various settings, and were cross-sectional in design (n=8). Of a total of 17 measures identified, 6 were originally developed for the general population and then adapted for PWD, and only 3 were validated for PWD. A majority of the studies were limited in sample size, generalizability, methodological rigor, and measure validation. More research is needed using diverse samples and rigorous study designs to develop valid screening and assessment tools for this population. Improving religious and spiritual measures could greatly impact public health by improving quality of life for millions of individuals suffering from dementia and their caregivers who carry a heavy burden.

